# Diarrhoeal outcomes in young children depend on diarrhoeal cases of other household members: a cross-sectional study of 16,025 people in rural Uganda

**DOI:** 10.1186/s12879-022-07468-2

**Published:** 2022-05-21

**Authors:** Farina L. Shaaban, Narcis B. Kabatereine, Goylette F. Chami

**Affiliations:** 1 Big Data Institute, Nuffield Department of Population Health, University of Oxford, Oxford, UK; 2 Division of Vector Borne Diseases and Neglected Tropical Diseases, Ministry of Health, Kampala, Uganda

**Keywords:** Diarrhoea, Sub-saharan Africa, WASH, Sanitation, Water, Household, Clustering, Community

## Abstract

**Background:**

There is a limited understanding of how diarrhoeal cases across other household members influence the likelihood of diarrhoea in young children (aged 1–4 years).

**Methods:**

We surveyed 16,025 individuals from 3421 households in 17 villages in Uganda. Using logistic regressions with standard errors clustered by household, diarrhoeal cases within households were used to predict diarrhoeal outcomes in young children. Regressions were adjusted for socio-demographic, water, sanitation, and hygiene (WASH), and ecological covariates. Selection bias for households with (1632/3421) and without (1789/3421) young children was examined.

**Results:**

Diarrhoeal prevalence was 13.7% (2118/16,025) across all study participants and 18.5% (439/2368) in young children. Young children in households with any other diarrhoeal cases were 5.71 times more likely to have diarrhoea than young children in households without any other diarrhoeal cases (95% CI: 4.48–7.26), increasing to over 29 times more likely when the other diarrhoeal case was in another young child (95% CI: 16.29–54.80). Diarrhoeal cases in older household members (aged ≥ 5 years) and their influence on the likelihood of diarrhoea in young children attenuated with age. School-aged children (5–14 years) had a greater influence on diarrhoeal cases in young children (Odds Ratio 2.70, 95% CI: 2.03–3.56) than adults of reproductive age (15–49 years; Odds Ratio 1.96, 95% CI: 1.47–2.59). Diarrhoeal cases in individuals aged ≥ 50 years were not significantly associated with diarrhoeal outcomes in young children (*P* > 0.05). These age-related differences in diarrhoeal exposures were not driven by sex. The magnitude and significance of the odds ratios remained similar when odds ratios were compared by sex within each age group. WASH factors did not influence the likelihood of diarrhoea in young children, despite influencing the likelihood of diarrhoea in school-aged children and adults. Households with young children differed from households without young children by diarrhoeal prevalence, household size, and village WASH infrastructure and ecology.

**Conclusions:**

Other diarrhoeal cases within households strongly influence the likelihood of diarrhoea in young children, and when controlled, removed the influence of WASH factors. Future research on childhood diarrhoea should consider effects of diarrhoeal cases within households and explore pathogen transmission between household members.

**Supplementary information:**

The online version contains supplementary material available at 10.1186/s12879-022-07468-2.

## Background

Diarrhoea-related mortality is most concentrated in children aged < 5 years, who account for an estimated 1.7 billion cases and over 500,000 deaths each year [1–4]. Diarrhoea, characterised as loose or watery stool, is often a symptom of enteric viral or bacterial infections causing gastroenteritis and several neglected tropical diseases [5, 6]. In infants (< 1 year) and young children (1–4 years), diarrhoea disrupts growth [7, 8]. Enteropathogens such as Shiga-toxin producing *Escherichia coli* and rotavirus cause diarrhoea and are commonly spread through the faecal-oral-route by contaminated water and food, or person-to-person contact [9]. Thus, safe water, sanitation, and hygiene (WASH) are at the centre of interventions to reduce the burden of diarrhoeal diseases. Despite global efforts to improve access to WASH as per Sustainable Development Goal 6, an estimated 88% of global diarrhoeal deaths remain linked to WASH factors, particularly in low-income regions [10, 11].

The relationships between WASH exposures and diarrhoeal prevalence have been studied extensively [12–16]. Most research on diarrhoeal outcomes has focused on children < 5 years of age, whereby WASH exposures are measured at the household level or as caregiver characteristics [9, 12, 15, 17]. However, findings regarding these relationships have been inconsistent and complex [18–20]. For instance, a study on diarrhoea in young children in Uganda found that individual-level WASH practices, such as drinking surface water without treatment, had no effect on childhood diarrhoeal outcomes, yet diarrhoeal prevalence was negatively associated with household-level scores of WASH [20].

Despite current studies [9, 12, 15, 17] in low-income countries on household-level exposures, the influence of diarrhoeal cases from other household members on the likelihood of diarrhoea in young children aged 1–4 years remains poorly understood. The aim of this study was to investigate how diarrhoeal cases across other household members influence the likelihood of diarrhoeal outcomes in young children while controlling for household-level WASH.

## Methods

### Study design and participants

This study was a secondary analysis of cross-sectional data collected using household surveys in November 2013 as part of a larger study on schistosomiasis (*Schistosoma mansoni*) [21, 22]. Seventeen villages within five kilometres of Lake Victoria were selected in Mayuge District, Uganda, as described by Chami et al. [23]. Lay surveyors conducted interviews with household heads, who may be female or male, and their spouses (if applicable) in all but 2.4% (87/3578) of households in the selected villages. Respondents reported medical and socio-environmental information for all members of the household aged ≥ 1 year, including themselves [21].

Inclusion criteria consisted of being (1) a current resident in the study village, as defined as spending ≥ 6 months of the year in the village and (2) aged ≥ 1 year at the time of survey. No exclusion criteria were applied to individuals. However, households were excluded if (1) no household member was in good health i.e., all adults were hospitalised or unable to complete daily tasks or (2) no sober person was available in the household to interview. Information was collected on 16,357 individuals. Complete information on all measured covariates was available for 16,025 (98.0%; 16 025/16 357) participants belonging to 3,421 (98.0%; 3421/3491) households. These individuals formed the final sample.

### Outcome

Participants were assigned positive diarrhoeal status if they had experienced at least one diarrhoeal episode in the three months prior to the survey. The World Health Organisation (WHO) definition of diarrhoea was used, which was the passage of three or more loose stools in a day [24]. Breastfeeding infants and children aged < 1 year were excluded from the analysis. Positive diarrhoeal status in children aged 1–4 years (henceforth referred to as young children) was the outcome of interest.

### Exposures

Diarrhoea in other household members was measured at the household level and excluded the young child of interest (diarrhoeal outcome). For the school-aged children and adults, to assess diarrhoeal cases within each age group, age was categorised into 5–14, 15–49 and ≥ 50 years to capture school-aged children, reproductive age groups, and older/elder populations and any diarrhoeal outcomes within that particular age group for a household were defined as binary variables [21]. A binary indicator for whether there was another young child with diarrhoea was coded as one if there were at least two young children (so at least one more than the child of interest) in the same household with diarrhoea.

### Covariates

Twenty-four candidate covariates were considered, including socio-demographic, WASH, and ecological variables. Age was examined to the nearest year, including 70 children younger than one who were coded as one year. A binary variable denoting sex was equal to one for female participants. The highest level of education attained by anyone in the household was an ordinal measure including no formal education (level 0), primary education (1–7), secondary education (8–13), and higher education (14 = diploma, 15 = some university, and 16 = completed university). Binary variables identified households that were part of the village majority tribe, had a Muslim household head, when the household head owned the home (otherwise rented), and when anyone in the household had social status. A household had social status if any household member was a health team member, local government official, or community leader within their village. Household size was measured as the number of individuals aged one year or older in the household. Village residence was measured as the years the household has been settled in the village. Household electricity was represented as a binary variable and a home quality score which ranged from 4 to 12 was constructed by summing floor, wall, and roof scores. Each material score ranged from 1 to 4 in order of the quality of materials for each part of the home, as described by Chami et al. [21]. Home quality score represented a general measure of socioeconomic status. In separate models, to investigate the possibility of young children eating dirt within a home with a mud floor, a binary variable for floor was investigated and coded to one if the floor was made of mud.

WASH variables were defined using the WHO/UNICEF Joint Monitoring Programme safe WASH definitions [25]. At the household level, the WASH variables included availability of at least 20 L of water per person, purification of drinking water, use of improved drinking water source, and availability of improved sanitation at home (see Additional file 1: Table S1 for more detail). At the village level, WASH variables included the availability of at least one working public tap or latrine in the village.

Ecological variables were measured at the village level. These included the presence of a rice paddy in the village, distance from the village centre to the lake (if > 0.5 km), total roads (if ≥ 3 roads), access to the lake (if had a beach or a boat landing site at Lake Victoria), total number of homes and average distance in metres between households in the same village. For total number of homes, a categorical variable was used with < 100 homes as the base category and other categories of 100–199, 200–299, and ≥ 300 homes.

### Statistical analyses

All analyses were conducted on Stata version 16.1. Selection of covariates for regression models was done using likelihood ratio tests (LRT) with *P* < 0.05. To predict diarrhoeal outcomes in young children using household diarrhoeal cases, we ran logistic regression models adjusted for the effects of relevant covariates (LRT *P* < 0.05). To uncover more detailed effects of diarrhoeal cases within households on the likelihood of diarrhoea in young children, the exposure variable was redefined into specific categories. These categories indicated first the age group only then the age group and sex of the other household members with diarrhoea.

All regression models accounted for household clustering of outcomes using clustered (robust) standard errors at the household level. The variance inflation factor (VIF) was calculated for each regression model to test for multicollinearity between independent variables. From pairs of collinear covariates (VIF ≥ 10), the more relevant covariate as informed by literature was selected for inclusion [26]. Model fit was assessed using 10-fold cross-validation [27].

To assess the risk of selection bias, as only a subset of households had children younger than five years (47.7% 1632/3421), characteristics of the study population were compared across included and excluded households using T-tests, Pearson χ² tests, and Wilcoxon rank sum tests as appropriate.

## Results

### Participant characteristics

Table 1 presents the characteristics of study participants and their households. Participants were evenly split by sex (Female: 50.5%, 8091/16,025) and had an average age of 22.6 (SD 16.3). There were 14.8% (2368/16,025) of participants who were young children. Across households with all participants (16,025), households had a median of six total members aged ≥ 1 year (Interquartile range (IQR) 4–8). The median level of highest education attained among any household member was the completion of primary school (7, IQR 6–9). Only 15.2% (2437/16,025) of individuals lived in households with improved (adequate) sanitation and 40.8% (6531/16,025) of individuals belonged to households that purified drinking water. A vast majority of the study population (81.2%, 13,010/16,025) lived in homes with mud floors.

**Table 1:** Characteristics of included versus excluded participants

n (%) or median (IQR)	All	Included^a^	Excluded^a^	P-value^b^
	n = 16025	(n = 9507)	(n = 6518)	
Diarrhoeal prevalence				
Overall prevalence	2188 (13.7)	1413 (14.9)	775 (11.9)	< 0.01
Prevalence by age				
1–4	439 (18.5)	439 (18.5)	0 (0.0)	
5–14	768 (13.6)	466 (14.7)	302 (12.2)	< 0.01
15–49	852 (12.4)	471 (13.1)	381 (11.7)	0.07
≥ 50	129 (11.1)	37 (10.0)	92 (11.7)	0.38
Socio-demographic characteristics				
Age (excluding < 5 years), mean (SD)^c^	*22.6 (16.3)*	*20.8 (14.6)*	*24.6 (17.8)*	< 0.01
Female	8091 (50.5)	4912 (51.7)	3179 (48.8)	< 0.01
Education, highest level attained in household	*7 (6–9)*	*7 (6–9)*	*7 (5–9)*	< 0.01
Household in village majority tribe	6381 (39.9)	3826 (40.2)	2555 (39.2)	0.18
Muslim household head	5011 (31.8)	3323 (35.0)	1688 (25.9)	< 0.01
Household w/ social status in village	1782 (11.1)	1000 (10.5)	782 (12)	< 0.01
Household size^d^	*6.0 (4–8)*	*7.0 (5–9)*	*5.0 (3–7)*	< 0.01
Years household settled in village	*13 (6–22)*	*11 (5–20)*	*15 (8–25)*	< 0.01
Household has electricity	998 (6.2)	546 (5.7)	452 (6.9)	< 0.01
Home quality score	*6 (3–9)*	*6 (3–9)*	*6 (3–9)*	0.03
Home has mud floor	13,010 (81.2)	7888 (83.0)	5122 (78.6)	< 0.01
Home owned by household head^e^	14,279 (89.6)	8405 (88.8)	5874 (90.7)	< 0.01
WASH characteristics				
20 L water available per household member	9827 (61.3)	5859 (61.6)	3968 (60.9)	0.338
Household purifies drinking water	6531 (40.8)	3821 (40.2)	2710 (41.6)	0.08
Improved drinking water	10 989 (68.6)	6528 (59.4)	4451 (40.6)	0.76
Improved sanitation	2437 (15.2)	1472 (15.5)	965 (14.8)	0.24
Public tap	10 675 (66.6)	6071 (63.9)	4604 (70.6)	< 0.01
Public latrine	4241 (26.5)	2655 (27.9)	1586 (24.3)	< 0.01
Ecological characteristics				
Rice paddy	11 925 (74.4)	7140 (75.1)	4785 (73.4)	0.02
Distance to Lake > 0.50 km	9023 (56.3)	5094 (53.6)	3929 (60.3)	< 0.01
Landing site or beach in village	12,490 (77.9)	7328 (77.1)	5162 (79.2)	< 0.01
≥ 3 roads in village	7821 (48.8)	4888 (51.4)	2933 (45.0)	< 0.01
Total homes				< 0.01
<100	293 (1.8)	222 (2.3)	71 (1.1)	
100–199	7448 (46.5)	4665 (49.1)	2783 (42.7)	
200–299	3921 (24.5)	2212 (23.3)	1709 (26.2)	
≥300	4363 (27.2)	2408 (25.3)	1955 (30.0)	
Mean distance (metres) btw households in same village	*376.0 (335.3-470.2)*	*376.0 (335.3-505.4)*	*376.0 (335.3-470.2)*	0.76

Italic values represent the median and IQR, whereas non-italic values are counts and percentages

^a^Of the 16,025 participants in 3421 households, 9507 (59.3%) lived in 1632 (47.7%) households with a young child (1–4 years) and were included in analyses, and 6518 (40.7%) lived in 1789 (52.3%) households without a young child and were excluded in analyses

^b^Pearson χ² and Wilcoxon rank sum tests comparing included and excluded participants

^c^Excluded participants. 13,657; 7139; 6518 individuals aged five years and older for the full study population, included only, and excluded only, respectively

^d^Measured in number of individuals aged ≥ 1 year in the household (n = 16 025)

^e^Home ownership has 15,944; 14,279; 1665 individuals for the full study population, included only, and excluded only, respectively, due to missing observations

### Diarrhoeal prevalence by age groups

**Fig. 1:**
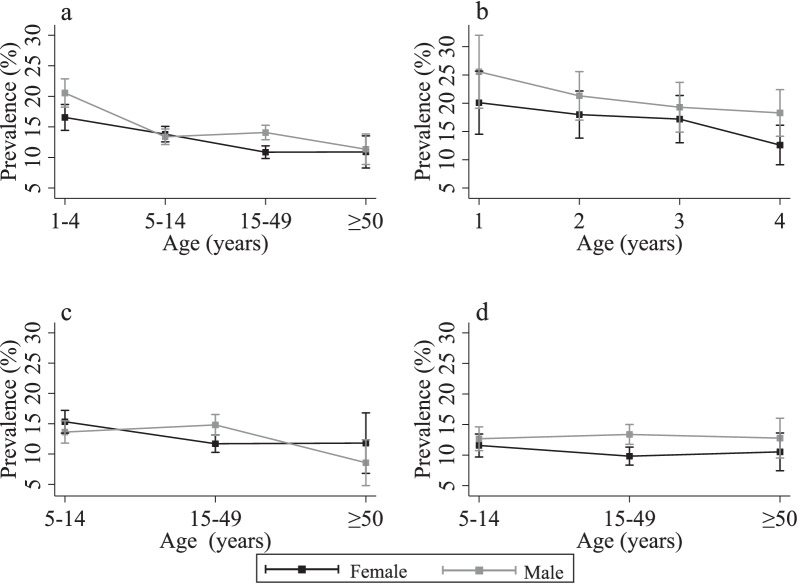
Prevalence of diarrhoea by age groups and sex. The squares denote average prevalence of diarrhoea and the vertical bars represent the corresponding 95% confidence intervals. **a** Diarrhoeal prevalence in the study population by sex. **b** Diarrhoeal prevalence in young children (aged 1–4 years) by sex. **c** Diarrhoeal prevalence for all age groups by sex where households had young children. **d** Diarrhoeal prevalence for all age groups by sex where households did not have young children

Figure 1 shows diarrhoeal prevalence by age and sex. In young children, diarrhoeal prevalence was 18.5% (439/2368) (Fig. 1a). Among young children, the prevalence of diarrhoea was highest in one-year-olds (22.7%; 85/375) and subsided with age to a prevalence of 15.4% (106/688) among children aged four years (Fig. 1b). Diarrhoeal prevalence across the entire study population was 13.7% (2118/16,025) (Table 1). In participants aged 5–14 years diarrhoeal prevalence was 13.6% (768/5647), 12.4% (852/6852) in participants aged 15–49 years, and 11.1% (129/1158) in participants aged ≥ 50 years. Diarrhoeal prevalence was negatively associated with age (χ² *P* < 0.001). A total of 33.6% (795/2368) of young children belonged to a household where someone else in the household had diarrhoea regardless of the diarrhoeal status of the young child of interest.

### Differences in households with and without young children

The 6518 (40.7%) excluded participants systematically differed from the 9507 (59.3%) included participants (Table 1). For households with young children, compared to households without young children, diarrhoeal prevalence was higher (14.9%; 1413/9057 versus 11.9%; 775/6518, χ² *P* < 0.01) (Fig. 1c, d), there was lower access to public taps (63.9%; 6071/9507 versus 70.6%; 4604/6518, χ² *P* < 0.01) and higher access to public latrines (27.9%; 2655/9507 versus 24.3%; 1586/6518, χ² *P* < 0.01). Households with young children (Median 7, IQR 5–9) were larger than households without young children (Median 5, IQR 3–7, χ² *P* < 0.01).

### Predictors of diarrhoea in young children

Selected covariates included age, sex, improved household drinking water, availability of a public tap and latrine, presence of a rice paddy, distance to the lake, total roads, and total homes. Average distance between households was also selected (LRT *P* < 0.01), but later excluded from regression models as it introduced multicollinearity (VIF > 10).

**Fig. 2:**
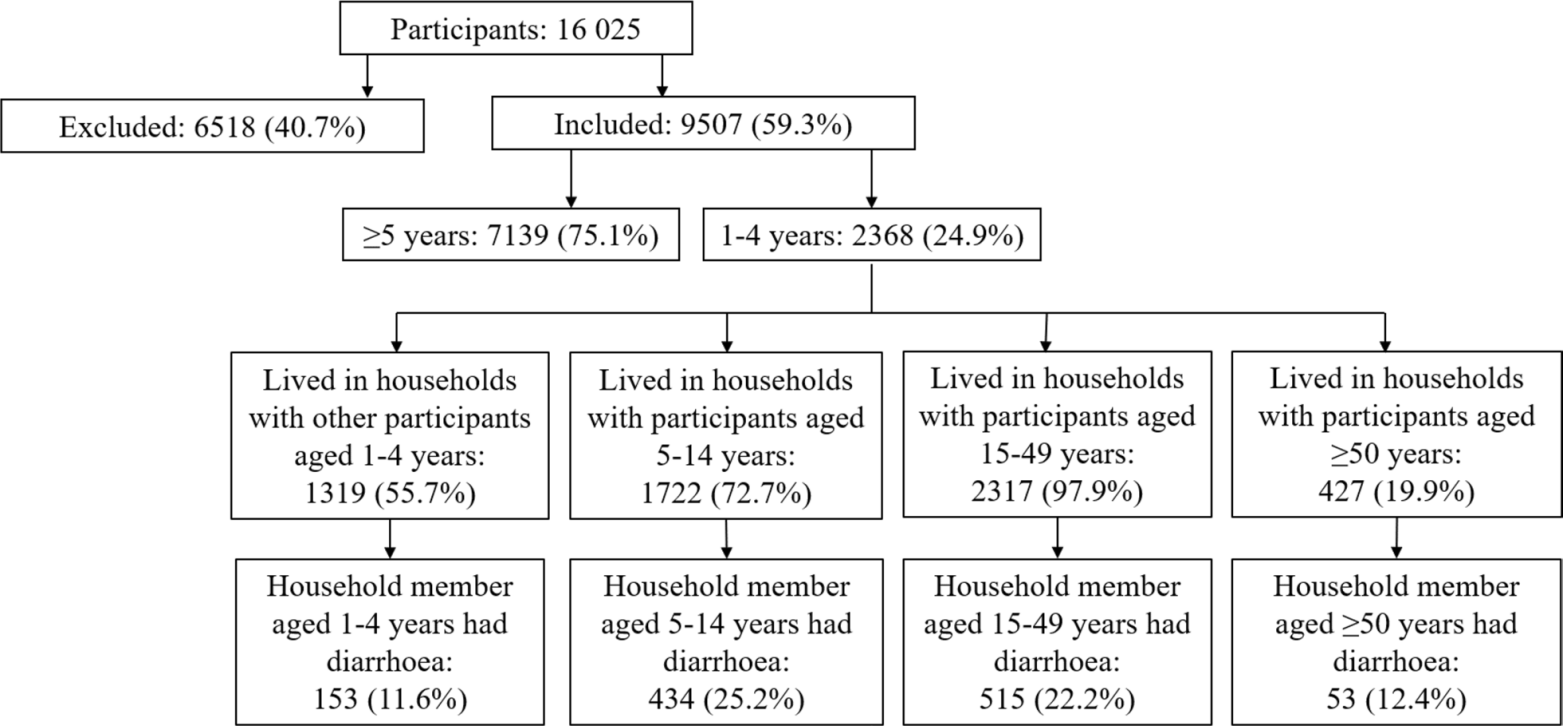
Distribution of young children across categories of household diarrhoeal cases. Participants were divided into included individuals who lived in households with young children (aged 1–4 years) and excluded individuals who lived in households without young children. Young children lived in households with members of other age groups. The lowest level of the flowchart illustrates the proportion of young children exposed to cases of diarrhoea within their households

**Fig. 3:**
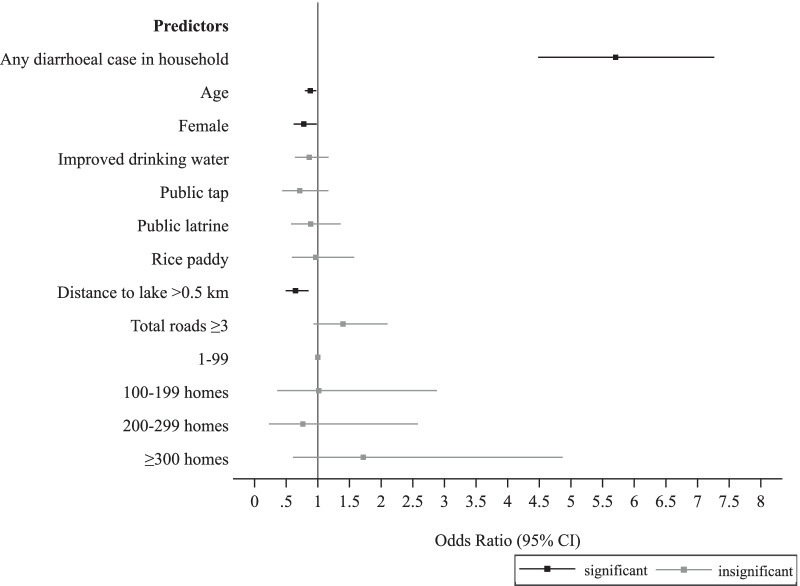
Predictors of diarrhoea in young children (aged 1–4 years). Odds ratios (squares) and corresponding 95% confidence intervals from the model reported in Additional file 1: Table S2 are shown. Black represents effects for which *P* < 0.05 and grey represents effects for which *P* ≥ 0.05

Figure 2 presents the distribution of young children across the exposure of interest. Figure 3 presents the predictors of diarrhoeal outcomes in young children. The estimates of the regression model are summarised in Additional file 1: Table S2. Over 57.52% of the variation of diarrhoeal outcomes in young children was explained within households (unadjusted intraclass clustering coefficient, 95% CI: 45.81–68.44). After adjusting for selected covariates (Additional file 1: Table S2), young children in households with other diarrhoeal cases were 5.71 times more likely to report diarrhoea compared to young children in households with no other diarrhoeal cases (95% CI: 4.48–7.26, Fig. 3). Each yearly increase in age resulted in a 12% lower likelihood of diarrhoea among young children (95% CI 0.80–0.98). Female children (1–4 years) were 22% less likely to have diarrhoea compared to male children of this age group (95% CI: 0.62–0.98). Young children in villages located > 0.5 km from Lake Victoria were 35% (95% CI: 0.49–0.85) less likely to have diarrhoea compared to young children in villages within 0.5 km of Lake Victoria (Fig. 3). WASH covariates either were not selected or were not associated with diarrhoea in young children (Fig. 3).

**Fig. 4:**
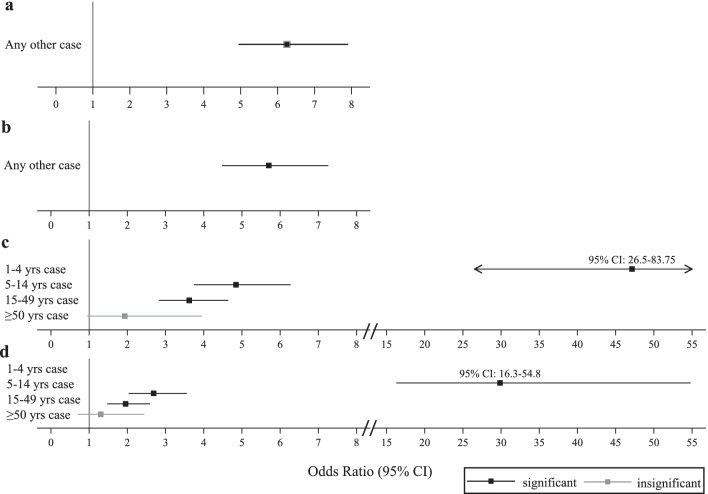
Effects of household diarrhoeal cases by age group. Odds ratios (squares) and corresponding 95% confidence intervals from models reported in Additional file 1: Tables S2 and S3 are shown. Black represents effects for which *P* < 0.05 and grey represents effects for which *P* ≥ 0.05. **a** and **b** Represent the effects of any other diarrhoeal case within the household on diarrhoeal outcomes in young children (aged 1–4 years) in unadjusted and adjusted regressions, respectively. **c** and **d** Present the effects of diarrhoeal cases across other household members belonging to different age groups in unadjusted and adjusted regressions, respectively

The effects of diarrhoeal cases across other household members on diarrhoeal outcomes in young children remained when the exposures were redefined by age group (Additional file 1: Table S3). Compared to households where no other young children had diarrhoea, young children in households including another young child with diarrhoea had 29.88 times higher likelihood of diarrhoea (95% CI: 16.29–54.80, Fig. 4). The effects of household diarrhoeal cases on diarrhoea in young children attenuated at older age groups. Young children in households where members aged 5–14 years had diarrhoea were 2.69 (95% CI: 2.03–3.56) times more likely to have diarrhoea when compared to young children without other household members aged 5–14 years with diarrhoea (Fig. 4). Similarly, young children with household members aged 15–49 years with diarrhoea were 1.96 (95% CI: 1.47–2.59) times more likely to have diarrhoea when compared to young children without other household members aged 15–49 years with diarrhoea. Diarrhoeal cases in the elderly (aged ≥ 50 years) were not associated with diarrhoeal cases in young children (Odds ratio 1.31, 95% CI: 0.70–2.44).

Notably, the magnitude of the effects of diarrhoeal cases in female household members aged 15–49 years, assumed to be the caregivers of young children, did not substantially differ from the size of the effects of diarrhoeal cases in other household members aged ≥ 5 years (Additional file 1: Table S4). Children in households with females aged 15–49 years with diarrhoea were 1.81 times more likely to have diarrhoea than children in households where females aged 15–49 years did not have diarrhoea (95% CI: 1.26–2.60). This effect fell within the confidence intervals of estimated effects of diarrhoeal cases in household members of other age groups and sex (Additional file 1: Table S4).

### Predictors of diarrhoea in older children and adults

Selected covariates for predicting diarrhoeal outcomes in older children and adults, excluding the young children, are presented in Additional file 1: Tables S5 and S6. Notably, educational attainment and improved sanitation were both correlated with outcomes in older children, but not in outcomes in young children (Additional file 1: Tables S2, S5). Each additional year of educational attainment, as measured by the highest level of education in the household, was associated with a 6% less likely chance of an individual aged five years or older having diarrhoea (95% CI 0.92–0.97). Oddly, improved sanitation in the household was positively associated with the likelihood of diarrhoea when compared to households without improved sanitation. An individual in a home with a mud floor was 1.36 times more likely to have diarrhoea than an individual in a home with a concrete, wood, or plastic floor (Table S6, 95% CI 1.09–1.70). Notably, models for young children were a much better fit than those constructed for adults (10-fold cross-validated area under Receiver Operating Curves of 0.86 for young children versus 0.64 for older children and adults).

## Discussion

Diarrhoea is a leading cause of morbidity and mortality in Uganda [12, 17]. Enteric infections that cause diarrhoea and their transmission patterns from person-to-person or via the faecal-oral-route are well understood. Yet, to our knowledge, we conducted the first investigation of patterns of diarrhoea within households and their influence on diarrhoeal outcomes in young children (aged 1–4 years). Analyses were conducted on 2368 young children from a survey of 16,025 individuals, aged ≥ 1 year, belonging to 3,421 households in 17 villages along the shores of Lake Victoria in Uganda. We found that diarrhoeal outcomes in young children were associated with diarrhoeal cases across other household members, sex, and distance from the village centre to the lake. Over 57% of the variation in diarrhoeal outcomes in young children was explained by factors within the household.

In this study, diarrhoeal prevalence in young children was 18.5%, slightly lower than the 20% national average presented in the Uganda Demographic and Health Survey in 2016 [28]. This may be due to the exclusion of infants in the current study. Although diarrhoeal prevalence subsided with age, it did not fall below 10% for any age group and was found to be 13.7% in the wider community. We found that diarrhoeal cases across other household members strongly influenced the likelihood of diarrhoea in young children. These findings suggest that the burden of diarrhoea at the household level puts at risk the most susceptible individuals within the household, young children. The effects of household diarrhoeal cases on diarrhoea in young children was observed across all age groups of other household members except those aged ≥ 50 years. The failure to detect an association in this age group may be the result of few young children in households with members aged ≥ 50 years and even fewer in households where members aged ≥ 50 years had diarrhoea. By using clustered standard errors by households and adjusting for household size, our findings suggest independent influences of diarrhoeal cases within households. Additionally, our findings cannot be explained by a lack of insufficient exposures (other diarrhoeal cases) in households with young children.

Surprisingly, investigating the effects of diarrhoeal cases across age groups and sex suggested no overwhelming influence of diarrhoeal cases in women of reproductive age (15–49 years; the most likely caregiver) on diarrhoeal outcomes in young children. Cases reported by females aged 15–49 years had similar odds ratios associated with the likelihood of diarrhoea in young children in comparison to those reported by other household members aged ≥ 5 years. This finding may explain why in previous studies no evidence was found for caregiver-targeted water and sanitation interventions and their ability to reduce childhood diarrhoea [19, 20]. Future research is needed to understand if caregiver-to-child transmission is occurring disproportionately in the household when compared to other members aged ≥ 5 years.

Most importantly, our findings identify diarrhoeal cases in other young children in the household to be the strongest predictor for diarrhoeal outcomes in young children. A study conducted in Tanzania estimated that young children ingested more faecal matter through hand-to-mouth contact than through drinking contaminated water [29]. The young children in our study might share specific transmission pathways which are not directly attributable to conventional household exposures such as WASH factors. In our study, little can be said about the effects of limited access to soap as well as poor hygiene practice, as they were not measured in this study, which is a limitation of the analysis. Promotion of caregiver-personal hygiene, as well as environmental hygiene in households, has been found to reduce diarrhoeal outcomes in young children [30, 31]. Furthermore, a systematic review found that faecal contamination on hands of children is a predictor of childhood diarrhoea [32]. Interactions, such as play, between young children and fomites paired with increased hand-to-mouth contact, may explain the large increase found here in the likelihood of diarrhoea in young children associated with household diarrhoeal cases in other young children. Our findings suggest that future research on the mechanisms of direct transmission among young children is needed. Interventions also could be investigated that include effects of different sanitary and hygiene materials for children; general household hygiene with respect to how young children interact with their environment and one another; as well as caregiver personal hygiene while caring for children.

We found that the influence of diarrhoeal cases of other household members on the likelihood of diarrhoea in young children attenuated with age. Odds ratios declined 10-fold from that observed for other young children (29.88) versus other school-aged children (2.69). A smaller decline of 73% was observed from school-aged children (2.69) to individuals of reproductive age (1.96). The odds ratio for the elderly was insignificant. Future research should investigate whether bigger gaps in age translate into differences in pathogen exposure and, in turn, whether this difference explains why older age groups are less likely to influence the likelihood of diarrhoea in young children. The Global Multicentre Enteric Study (GEMS) already shows that the aetiological agents of diarrhoea differ even among young children [33]. Our study might suggest that the aetiological agents of diarrhoea potentially still could be more similar among young children than when young children are compared to older age groups. Additional research is needed to investigate how the type and diversity of enteric pathogens vary among individuals of all ages.

The transmission and burden of diarrhoeal diseases have commonly been attributed to poor water, sanitation, and hygiene conditions [34]. Yet, the impacts of WASH interventions in rural settings of low-income countries are limited, and rarely cater to entire populations [34, 35]. For example, definitions of improved sanitation infrastructure include facilities not utilised by young children [25]. Our analyses have highlighted a challenge of WASH interventions in reducing diarrhoeal prevalence by presenting no evidence of their effect on the likelihood of diarrhoea in young children. It is unlikely due to underfitting of the model, i.e. specifically missing WASH covariates, as our model was highly predictive of diarrhoeal outcomes in young children. Similar to our results, a study in Kenya found that water treatment, improved household sanitation, and improved access to handwashing resources did not reduce diarrhoeal prevalence in young children although adherence in the targeted group (caregivers) was high [19]. Our study suggests that these findings may be due to the lack of child-targeted interventions.

Importantly, we found that within the study population, households with young children differed from households without young children. On average, participants in households with a young child had higher diarrhoeal prevalence, belonged to larger households, and were more likely to live in villages without a working public tap. This potential selection bias in the study of young children with diarrhoea has not been addressed elsewhere despite the existence of large-scale epidemiological studies [36, 37]. Future research investigating household patterns of diarrhoea should recognise that households with young children may not be representative of the general population with their access to WASH. This key selection bias, given that diarrhoeal studies focus on young children and interventions focus on community-level infrastructure, may help explain why some WASH interventions have repeatedly failed to reduce the burden of diarrhoeal outcomes in sub-Saharan Africa.

One limitation of this analysis is the use of self-reported diarrhoeal outcomes by household heads and, if applicable, their spouses. The outcome was dependent on the knowledge of respondents on the health-related experiences of all members of their household. Unlike clinical definitions of diarrhoeal outcomes, the self-reports—to avoid recall bias—did not provide information about the severity of diarrhoea. Outcomes in older members of the household might be underestimated as household respondents may not be aware of them, which is especially true for milder diarrhoeal episodes. However, as described by Chami et al. [21], the study context lacks electronic medical records, individuals have poor healthcare-seeking behaviours, and self-reports of diarrhoea remain the primary method of diagnosis within local health centres. Self-reports are commonly used in other community-based studies to survey diarrhoea [18–20]. We used clustered standard errors to account for correlations in diarrhoeal reports within households although there may still be a difference in reporting behaviours across households. Another limitation of using a cross-sectional study design is that this analysis cannot address reverse causality. Existing studies [12–16] overwhelmingly suggest that the mode of transmission is from caregiver to young child rather than from young child to caregiver. However, future studies should investigate household diarrhoeal outcomes prospectively to untangle the directionality of this relationship and progress towards causal inference.

## Conclusions

Diarrhoeal cases across other household members were associated with a higher likelihood of diarrhoeal outcomes in young children in rural Uganda. We found no support for an association between WASH factors and diarrhoea in young children. These findings reveal a potential limitation of untargeted WASH interventions. Future research should consider the influence of cases of diarrhoea across household members, in particular other young children, on childhood diarrhoea to plan interventions aimed at reducing diarrhoeal prevalence in young children.

## Supplementary information

**Additional file 1: TableS1. **Household level WASH covariates. **TableS2**. Predictors of diarrhoeal outcomes in young children (Model 1). **TableS3**. Predictors of diarrhoeal outcomes in young children (Model 2). **TableS4**. Predictors of diarrhoeal outcomes in young children (Model 3). **TableS5**. Predictors of diarrhoeal outcomes in older children and adults (Model 1). **TableS6**. Predictors of diarrhoeal outcomes in older children and adults (Model 2).

## Data Availability

The datasets generated and/or analysed during the current study are not publicly available due to ethics restrictions, but are available from the corresponding author on reasonable request.

## Abbreviations

WHO: World Health Organization
UNICEF: United Nation’s Children Fund
WASH: Water, sanitation, and hygiene
LRT: Likelihood ratio test
VIF: Variation inflation factor
IQR: Interquartile range
SD: Standard deviation
GEMS: Global Enteric Multicenter Study
MAL-ED: Etiology, Risk Factors, and Interactions of Enteric Infections and Malnutrition and the Consequences for Child Health
