# Supplementary material for: Diarrhoeal outcomes in young children depend on diarrhoeal cases of other household members: a cross-sectional study of 16,025 people in rural Uganda
Source: BMC Infect Dis. 2022 May 21;22:484. doi: 10.1186/s12879-022-07468-2 (PMC9123767; doi:10.1186/s12879-022-07468-2)
Supplement: Supplementary file 1 — Additional file 1: TableS1. Household level WASH covariates. TableS2. Predictors of diarrhoeal outcomes in young children (Model 1). TableS3. Predictors of diarrhoeal outcomes in young children (Model 2). TableS4. Predictors of diarrhoeal outcomes in young children (Model 3). TableS5. Predictors of diarrhoeal outcomes in older children and adults (Model 1). TableS6. Predictors of diarrhoeal outcomes in older children and adults (Model 2). [file 12879_2022_7468_MOESM1_ESM.docx]

**Additional File 1 for** “Diarrhoeal outcomes in young children depend on diarrhoeal cases of other household members: A cross-sectional study of 16 025 people in rural Uganda”

**Table of contents**

[**Table S1.** Household level WASH covariates 2](#_Toc98775213)

[**Table S2.** Predictors of diarrhoeal outcomes in young children (Model 1) 3](#_Toc98775214)

[**Table S3.** Predictors of diarrhoeal outcomes in young children (Model 2) 4](#_Toc98775215)

[**Table S4.** Predictors of diarrhoeal outcomes in young children (Model 3) 5](#_Toc98775216)

[**Table S5.** Predictors of diarrhoeal outcomes in older children and adults (Model 1) 6](#_Toc98775217)

[**Table S6.** Predictors of diarrhoeal outcomes in older children and adults (Model 2) 7](#_Toc98775218)

# **Table S1.** Household level WASH covariates

| **WASH exposure** | **Definition** | |
| --- | --- | --- |
| Water availability | **Sufficient** Household has access to ≥20 litres of water per person per day for consumption and personal hygiene | **Insufficient** Household has access to <20 litres of water per person per day for consumption and personal hygiene |
| Home water treatment | **Household treats water** method reported:  Bleaching,  boiling,  stand and settle,  straining  washing jerry cans with soap,  filtering,  solar method or  other methods | **Household does not treat water** No method reported |
| Drinking water | **Improved** Reported source:  Protected spring,  Bore hole,  Public tap,  Rainwater or  Bottled water | **Unimproved** Reported source:  Unprotected spring,  Lake,  Stream or   Other source |
| Sanitation | **Improved** Reported facility:  Flushable toilet or  private latrine and   facility not shared between households | **Unimproved** Reported facility:  Bucket latrine,  open latrine,  lake,  other facility,  no facility or  facility shared between households |

These WASH covariates were measured at the household level and coded equally for all members of the household

# **Table S2.** Predictors of diarrhoeal outcomes in young children (Model 1)

| **Predictors** | **Odds ratio** | **Clustered SE ^a^** | **95% CI** | | **P-value** |
| --- | --- | --- | --- | --- | --- |
| Any other diarrhoeal case in household | 5.705 | 0.703 | 4.481 | 7.263 | <0.001 |
| Age (years) | 0.882 | 0.046 | 0.797 | 0.976 | 0.015 |
| Female | 0.779 | 0.092 | 0.618 | 0.982 | 0.035 |
| Improved drinking water | 0.864 | 0.134 | 0.638 | 1.170 | 0.345 |
| Public tap | 0.713 | 0.179 | 0.436 | 1.168 | 0.179 |
| Public latrine | 0.886 | 0.194 | 0.576 | 1.361 | 0.579 |
| Rice paddy | 0.964 | 0.241 | 0.591 | 1.573 | 0.883 |
| Distance to lake >0.5 km | 0.647 | 0.091 | 0.491 | 0.853 | 0.002 |
| Total roads ≥3 | 1.398 | 0.290 | 0.931 | 2.101 | 0.107 |
| 100-199 homes ^b^ | 1.015 | 0.540 | 0.357 | 2.882 | 0.978 |
| 200-299 homes | 0.764 | 0.475 | 0.226 | 2.581 | 0.665 |
| ≥300 homes | 1.717 | 0.913 | 0.605 | 4.868 | 0.309 |
| Constant | 0.208 | 0.126 | 0.063 | 0.684 | 0.010 |

Total number of observations =2368 (including 439 outcomes) . The distance between households in meters was excluded due to collinearity (VIF>10.)

10-Fold cross-validated mean area under the ROC curve =0.860

^a^ 1632 household clusters

^b^ Joint p-value =0.017

# **Table S3.** Predictors of diarrhoeal outcomes in young children (Model 2)

| **Predictors** | **OR** | **Clustered SE** ^a^ | | **95% CI** | | | **P-value** | |  |
| --- | --- | --- | --- | --- | --- | --- | --- | --- | --- |
| Another young child w/ diarrhoea | 29.876 | | 9.247 | | 16.288 | 54.799 | | <0.001 | |
| Member aged 5-14 years w/ diarrhoea | 2.692 | | 0.385 | | 2.034 | 3.561 | | <0.001 | |
| Member aged 15-49 years w/ diarrhoea | 1.955 | | 0.281 | | 1.474 | 2.592 | | <0.001 | |
| Member aged ≥50 years w/ diarrhoea | 1.306 | | 0.417 | | 0.698 | 2.443 | | 0.403 | |
| Age (years) | 0.893 | | 0.052 | | 0.797 | 1.001 | | 0.053 | |
| Female | 0.847 | | 0.107 | | 0.662 | 1.084 | | 0.187 | |
| Improved drinking water | 0.865 | | 0.132 | | 0.641 | 1.167 | | 0.342 | |
| Public tap | 0.710 | | 0.177 | | 0.436 | 1.156 | | 0.168 | |
| Public latrine | 0.970 | | 0.223 | | 0.619 | 1.521 | | 0.896 | |
| Rice paddy | 0.889 | | 0.215 | | 0.554 | 1.427 | | 0.626 | |
| Distance to lake >0.5 km | 0.636 | | 0.090 | | 0.482 | 0.840 | | 0.001 | |
| Total roads ≥3 | 1.270 | | 0.256 | | 0.856 | 1.884 | | 0.236 | |
| 100-199 homes ^b^ | 1.455 | | 0.712 | | 0.558 | 3.798 | | 0.443 | |
| 200-299 homes | 1.063 | | 0.619 | | 0.340 | 3.328 | | 0.916 | |
| ≥300 homes | 2.238 | | 1.085 | | 0.866 | 5.786 | | 0.096 | |
| Constant | 0.165 | | 0.095 | | 0.053 | 0.512 | | 0.002 | |

Total number of observations =2368 (including 439 cases). The distance between households in meters was excluded due to collinearity (VIF>10.)

10-Fold cross-validated mean area under the ROC curve =0.820

^a^ 1632 household clusters

^b^ Joint p-value = 0.011

# **Table S4.** Predictors of diarrhoeal outcomes in young children (Model 3)

| **Predictors** | **OR** | **Clustered SE** ^a^ | | **95% CI** | | | **P-value** | |  |
| --- | --- | --- | --- | --- | --- | --- | --- | --- | --- |
| Another young child w/ diarrhoea | 29.497 | | 9.181 | | 16.026 | 54.289 | | <0.001 | |
| Member aged 5-14 years w/ diarrhoea, female | 2.053 | | 0.341 | | 1.483 | 2.843 | | <0.001 | |
| Member aged 5-14 years w/ diarrhoea, male | 2.095 | | 0.377 | | 1.472 | 2.981 | | <0.001 | |
| Member aged 15-49 years w/ diarrhoea, female | 1.807 | | 0.335 | | 1.257 | 2.599 | | 0.001 | |
| Member aged 15-49 years w/ diarrhoea, male | 1.455 | | 0.254 | | 1.034 | 2.048 | | 0.032 | |
| Member aged ≥50 years w/ diarrhoea, female | 1.576 | | 0.539 | | 0.807 | 3.079 | | 0.183 | |
| Member aged ≥50 years w/ diarrhoea, male | 0.980 | | 0.540 | | 0.333 | 2.885 | | 0.971 | |
| Age (years) | 0.899 | | 0.052 | | 0.802 | 1.008 | | 0.069 | |
| Female | 0.843 | | 0.106 | | 0.658 | 1.079 | | 0.174 | |
| Improved drinking water | 0.899 | | 0.138 | | 0.665 | 1.216 | | 0.491 | |
| Public tap | 0.733 | | 0.178 | | 0.455 | 1.180 | | 0.200 | |
| Public latrine | 1.014 | | 0.233 | | 0.647 | 1.591 | | 0.952 | |
| Rice paddy | 0.883 | | 0.212 | | 0.551 | 1.414 | | 0.603 | |
| Distance to lake >0.5 km | 0.661 | | 0.094 | | 0.500 | 0.874 | | 0.004 | |
| Total roads ≥3 | 1.274 | | 0.254 | | 0.862 | 1.884 | | 0.224 | |
| 100-199 homes ^b^ | 1.447 | | 0.713 | | 0.552 | 3.798 | | 0.453 | |
| 200-299 homes | 1.018 | | 0.592 | | 0.325 | 3.185 | | 0.976 | |
| ≥300 homes | 2.112 | | 1.021 | | 0.818 | 5.450 | | 0.122 | |
| Constant | 0.164 | | 0.095 | | 0.052 | 0.512 | | 0.002 | |

Total number of observations =2368 (including 439 cases). The distance between households in meters was excluded due to collinearity (VIF>10.)

10-Fold cross-validated mean area under the ROC curve =0.708

^a^ 1632 household clusters

^b^ Joint p-value = 0.013

# **Table S5.** Predictors of diarrhoeal outcomes in older children and adults (Model 1)

| **Predictors** | **Odds ratio** | **Clustered SE ^a^** | **95% CI** | | **P-value** |
| --- | --- | --- | --- | --- | --- |
| Age (years) | 0.996 | 0.002 | 0.993 | 1.000 | 0.027 |
| Female | 0.872 | 0.045 | 0.789 | 0.964 | 0.008 |
| Education, highest level attained in household | 0.940 | 0.013 | 0.916 | 0.965 | <0.001 |
| Household in village majority tribe | 0.879 | 0.071 | 0.751 | 1.029 | 0.109 |
| Muslim household head | 1.097 | 0.092 | 0.930 | 1.293 | 0.272 |
| Household w/ social status in village | 0.891 | 0.140 | 0.654 | 1.212 | 0.461 |
| Years household settled in village | 0.997 | 0.003 | 0.991 | 1.003 | 0.363 |
| Home quality score | 0.976 | 0.012 | 0.952 | 1.000 | 0.055 |
| Household purifies drinking water | 1.086 | 0.089 | 0.925 | 1.275 | 0.313 |
| Improved drinking water | 0.801 | 0.079 | 0.661 | 0.972 | 0.024 |
| Improved sanitation | 1.359 | 0.153 | 1.090 | 1.693 | 0.006 |
| Public tap | 0.687 | 0.068 | 0.566 | 0.834 | <0.001 |
| Public latrine | 1.068 | 0.095 | 0.897 | 1.271 | 0.462 |
| Rice paddy | 0.921 | 0.125 | 0.706 | 1.201 | 0.543 |
| Distance to lake >0.5 km | 0.522 | 0.045 | 0.441 | 0.618 | <0.001 |
| Total roads ≥3 | 1.314 | 0.139 | 1.068 | 1.616 | 0.010 |
| Constant | 0.579 | 0.113 | 0.395 | 0.848 | 0.005 |

Diarrhoeal outcomes in all individuals, excluding the young children (aged 1-4 years), were investigated.

Total number of observations = 13657 (including 1749 outcomes).

Home ownership, distance between households in meters, and total homes in the village were excluded due to collinearity (VIF>10). Model 1 differs from Model 2 in that the former has the overall home quality score, and the latter has only the type of flooring.

10-Fold cross-validated mean area under the ROC curve =0.643

^a^ 3421 household clusters

# **Table S6.** Predictors of diarrhoeal outcomes in older children and adults (Model 2)

| **Predictors** | **Odds ratio** | **Clustered SE ^a^** | **95% CI** | | **P-value** |
| --- | --- | --- | --- | --- | --- |
| Age (years) | 0.996 | 0.002 | 0.993 | 1.000 | 0.029 |
| Female | 0.873 | 0.045 | 0.789 | 0.965 | 0.008 |
| Education, highest level attained in household | 0.941 | 0.013 | 0.917 | 0.966 | <0.001 |
| Household in village majority tribe | 0.866 | 0.070 | 0.739 | 1.014 | 0.075 |
| Muslim household head | 1.095 | 0.092 | 0.928 | 1.292 | 0.281 |
| Household w/ social status in village | 0.887 | 0.139 | 0.653 | 1.207 | 0.446 |
| Years household settled in village | 0.997 | 0.003 | 0.990 | 1.003 | 0.314 |
| Mud floor | 1.360 | 0.157 | 1.085 | 1.704 | 0.008 |
| Household purifies drinking water | 1.091 | 0.090 | 0.929 | 1.282 | 0.287 |
| Improved drinking water | 0.798 | 0.078 | 0.658 | 0.967 | 0.021 |
| Improved sanitation | 1.354 | 0.152 | 1.087 | 1.687 | 0.007 |
| Public tap | 0.693 | 0.068 | 0.571 | 0.840 | <0.001 |
| Public latrine | 1.096 | 0.097 | 0.922 | 1.303 | 0.299 |
| Rice paddy | 0.948 | 0.129 | 0.727 | 1.236 | 0.693 |
| Distance to lake >0.5 km | 0.509 | 0.044 | 0.428 | 0.604 | <0.001 |
| Total roads ≥3 | 1.274 | 0.134 | 1.038 | 1.565 | 0.021 |
| Constant | 0.380 | 0.086 | 0.244 | 0.591 | <0.001 |

Diarrhoeal outcomes in all individuals, excluding the young children (aged 1-4 years), were investigated.

Total number of observations = 13657 (including 1749 outcomes).

Home ownership, distance between households in meters, and total homes in the village were excluded due to collinearity (VIF>10). Model 1 differs from Model 2 in that the former has the overall home quality score, and the latter has only the type of flooring.

10-Fold cross-validated mean area under the ROC curve =0.644

^a^ 3421 household clusters
